# Hands and Health at Home: An Innovative Intergenerational Program for Meals on Wheels Clients

**DOI:** 10.1093/geroni/igaa057.023

**Published:** 2020-12-16

**Authors:** Meghan Gatward, Rachel Logue, Courtney Vanderlaan, Susan Brown

**Affiliations:** 1 University of Michigan, Ann Arbor, Michigan, United States; 2 Michigan Medicine, Ann Arbor, Michigan, United States

## Abstract

Homebound older adults represent an understudied population who are at greater risk of losing hand strength and manipulation skills that, in turn, can lead to increased disability and cognitive declines (Dayanidhi and Valero-Cuevas, 2014). The Hands and Health at Home program was developed through a partnership with the University of Michigan’s School of Kinesiology and Michigan Medicine’s Ann Arbor Meals on Wheels program to demonstrate the feasibility of an intergenerational approach to address unmet needs of Meals on Wheels recipients. Undergraduate movement science student trainers were paired with a client who they visited twice weekly for 5 weeks. Students received training, including mock training scenarios, from an interprofessional team with backgrounds in social work, nursing, and neurorehabilitation. Home training protocols were developed using commercially available games and occupational therapy tools with the aim of improving hand function and facilitating socialization. Pre- and post-assessments included hand strength and dexterity, and client-reported measures of physical function and self-efficacy. Feedback from clients and students was overwhelmingly positive with several students indicating that the experience had stimulated interest in pursuing gerontology careers. Changes in quantitative assessments were variable across clients although pinch strength increased significantly in the non-dominant hand (p<0.02) and was predictive of measures of self-efficacy (r=.78, p<0.02). To our knowledge, this pilot program is the first of its kind and demonstrates the value of an intergenerational approach aimed at improving quality of life in Meals on Wheels clients, and may be of benefit for other underserved older members of the community.

